# A viewpoint on ecological and evolutionary study of plant thermal performance curves in a warming world

**DOI:** 10.1093/aobpla/plac016

**Published:** 2022-04-13

**Authors:** Rachel Wooliver, Emma E Vtipilthorpe, Amelia M Wiegmann, Seema N Sheth

**Affiliations:** Department of Plant and Microbial Biology, North Carolina State University, Raleigh, NC 27695, USA; Department of Plant and Microbial Biology, North Carolina State University, Raleigh, NC 27695, USA; Department of Plant and Microbial Biology, North Carolina State University, Raleigh, NC 27695, USA; Department of Plant and Microbial Biology, North Carolina State University, Raleigh, NC 27695, USA

**Keywords:** Climate change, evolutionary ecology, genetic variation, plasticity, thermal adaptation, thermal niche, thermal performance curve, trade-off

## Abstract

We can understand the ecology and evolution of plant thermal niches through thermal performance curves (TPCs), which are unimodal, continuous reaction norms of performance across a temperature gradient. Though there are numerous plant TPC studies, plants remain under-represented in syntheses of TPCs. Further, few studies quantify plant TPCs from fitness-based measurements (i.e. growth, survival and reproduction at the individual level and above), limiting our ability to draw conclusions from the existing literature about plant thermal adaptation. We describe recent plant studies that use a fitness-based TPC approach to test fundamental ecological and evolutionary hypotheses, some of which have uncovered key drivers of climate change responses. Then, we outline three conceptual questions in ecology and evolutionary biology for future plant TPC studies: (i) Do populations and species harbour genetic variation for TPCs? (ii) Do plant TPCs exhibit plastic responses to abiotic and biotic factors? (iii) Do fitness-based TPCs scale up to population-level thermal niches? Moving forward, plant ecologists and evolutionary biologists can capitalize on TPCs to understand how plasticity and adaptation will influence plant responses to climate change.

## Teaser

Variation in plant performance across temperatures are illustrated with a thermal performance curve (TPC), where performance peaks at a benign temperature and decreases to zero as temperature becomes cooler or warmer. Thermal performance curves have been useful for understanding evolution and plasticity, especially in response to climate change. However, most plant TPCs in the literature are based on physiological traits of single leaves, rather than individual fitness which would more directly approximate how populations might respond to changes in temperature. We describe what has been done and necessary next steps that utilize TPCs to determine how plants will respond to climate change.

## Introduction

**Figure 1.**
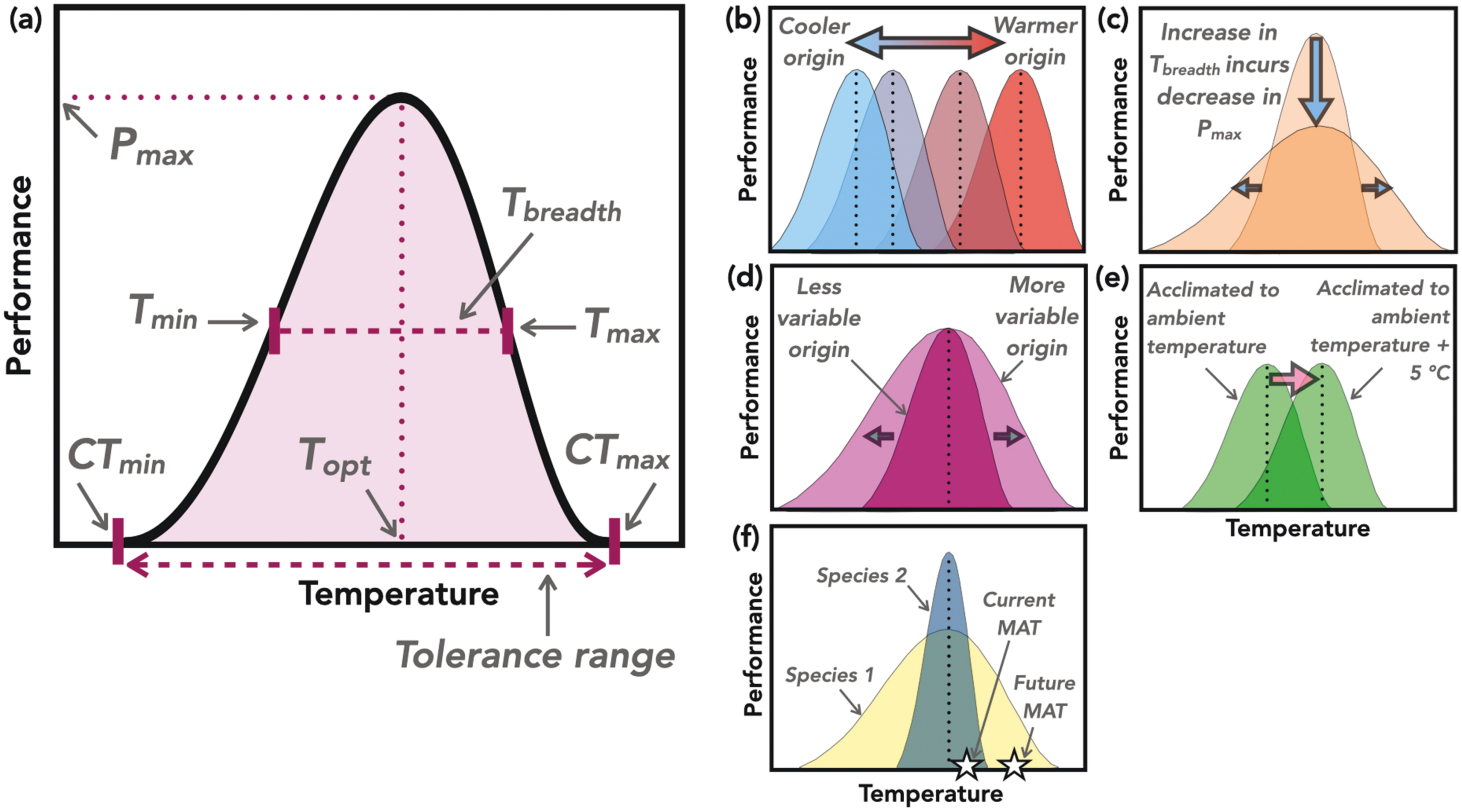
Illustration of a TPC and examples of applications of TPCs. (A) A TPC has a maximum performance (*P*_max_) at the thermal optimum (*T*_opt_), above and below which performance decreases. The temperatures at which performance reaches zero are the critical thermal minimum and maximum (CT_min_, CT_max_; the first way to quantify thermal limits), the span between which is the tolerance range. The range of temperatures across which performance reaches a certain percentage (usually 50 or 80 %) of maximum performance is the thermal performance breadth (*T*_breadth_), which is bounded by the thermal minimum and maximum (*T*_min_, *T*_max_; the second way to quantify thermal limits). Thermal performance curves are useful for assessing (B) evolutionary divergence, for example where thermal optimum, critical thermal minimum and critical thermal maximum of populations across a species geographic range have adaptively diverged across temperature gradients, indicating performance trade-offs between low and high temperatures; (C) specialist–generalist trade-offs, where an increase in thermal performance breadth comes at the cost of decreased maximum performance; (D) the climate variability hypothesis, where greater temperature variability induces the evolution of greater thermal performance breadth; (E) acclimation, for example where thermal optimum (shown with vertical dotted lines) is greater for an individual that has been acclimated to 5 °C above ambient temperature compared to an individual that has been acclimated to the ambient temperature; and (F) predictions of species responses to climate change. For example, generalist species whose broader TPCs encompass the predicted local increase in mean annual temperature (MAT; species 1) should fare well under warming climates, while specialist species whose narrower TPCs do not encompass the predicted local increase in temperature should be more vulnerable to a warming climate (species 2; Deutsch *et al.* 2008).

Temperature is among the most important global drivers of productivity and biodiversity (Mayhew *et al.* 2012; Moles *et al.* 2014; Reich *et al.* 2014). Likely due to being sessile, plants have evolved several ways to cope with a diversity of thermal environments, ranging from alpine and arctic tundra (Bliss 1962) to geothermal soils (Redman *et al.* 2002; Lekberg *et al.* 2012), and from continental regions with large temperature swings to thermally buffered coastal climates (Leinonen *et al.* 2009). Thermal adaptation has led to genetic differentiation in thermal performance traits within species (Angert *et al.* 2011; Gremer *et al.* 2020), and, in some cases, reproductive isolation with subsequent speciation (Keller and Seehausen 2012). Understanding the drivers of variation in thermal niches among plant populations, species and clades can inform assessments of vulnerability to climate change. To date, syntheses of thermal tolerance that include plants have focused primarily on thermal limits (which are the minimum and maximum temperatures that constrain necessary biological functions; Fig. 1A) of subindividual traits such as leaf-level photosynthesis (Lancaster and Humphreys 2020; Bennett *et al.* 2021; Geange *et al.* 2021). While thermal limits are valuable for predicting responses to climate change, ecological trade-offs between thermal performance traits (such as the cost of low performance that comes with a high range of tolerable temperatures; Angilletta *et al.* 2003) necessitate the use of a thermal performance curve (TPC) approach, which can estimate several thermal traits simultaneously (Huey and Stevenson 1979; Angilletta 2009).

Thermal performance curves are unimodal, continuous reaction norms of performance, defined as any metric of an organism’s ability to function, across a temperature gradient (Angilletta 2009; Fig. 1A). Unlike traits that are represented by a single number, traits that comprise TPCs are function-valued because they involve continuous mathematical functions (Kingsolver *et al.* 2001). Thermal performance curves are specific to performance metrics that reach a maximum value at a thermal optimum and decrease on either side of the thermal optimum towards the thermal limits. Thermal performance curve parameters such as thermal optimum and performance breadth (a measure of the width of the TPC; Fig. 1A) can constrain one another through genetic correlations and ecological trade-offs (Gilchrist 1996; Phillips *et al.* 2014). Thus, by simultaneously quantifying critical thermal limits, breadth and optimum, TPCs allow for more powerful tests of the evolutionary and ecological mechanisms that underlie thermal adaptation. Despite known trade-offs between thermal traits, only 5 % of plant studies that investigate photosynthetic thermal limits simultaneously quantify both upper and lower limits (Geange *et al.* 2021), and more studies of plant responses across broad temperature gradients are needed. Further, though there are numerous plant studies in the TPC literature, plants remain under-represented in syntheses of TPCs (Angilletta 2009; Sinclair *et al.* 2016), except for one meta-analysis examining how TPCs vary with level of biological organization (subindividual level in plants, individual-level in lizards and population-level in insects; Rezende and Bozinovic 2019). Still, our ability to use TPCs for evolutionary inferences is limited by the paucity of studies that estimate TPCs based on traits that approximate fitness components such as germination, survival and growth rate.

Here, we convey the value of fitness-based TPCs for addressing key questions in plant ecology and evolutionary biology. By fitness-based TPCs, we mean TPCs that are quantified through performance traits that are measured at the individual level or above (e.g. above-ground biomass or percent survival of different individuals across multiple temperatures). We do not discuss TPCs characterized by short-term, subindividual traits (e.g. photosynthesis) or the physiological or molecular mechanisms underlying such TPCs as these topics are already well-studied (Geange *et al.* 2021), but connecting such TPCs to genotype- and population-level TPCs is a crucial next step in unifying the plant TPC literature (Louthan *et al.* 2021). First, to illustrate the conceptual areas where TPCs hold particular promise, we highlight plant studies that use a TPC approach to test fundamental ecological and evolutionary hypotheses. Second, we outline three unanswered questions in ecology and evolutionary biology: (i) Do populations and species harbour genetic variation for TPCs? (ii) Do plant TPCs exhibit plastic responses to abiotic and biotic factors? (iii) Do fitness-based TPCs scale up to population-level thermal niches? Throughout our viewpoint, we describe how TPCs can enable predictions of plant responses to climate change.

## Past Use of TPCs to Address Key Questions in Plant Ecology and Evolution

### Hypotheses on TPC evolution

By approximating thermal niches, TPCs are central to understanding evolutionary and ecological dynamics across space and time. Using TPCs, we can test three major hypotheses in evolutionary ecology in the context of responses to climate change: the hot–cold trade-off, the specialist–generalist trade-off and associations between climate variability and niche breadth. First, if there are biochemical constraints to adapting to both low and high temperatures, there should be performance trade-offs between hot and cold environments (Fig. 1B; Kingsolver *et al.* 2004). Supporting this hypothesis, species lower and upper optimum germination temperatures (i.e. temperatures between which germination percentages are at least 95 %) significantly decrease towards the poles (Sentinella *et al.* 2020). More plant species are currently experiencing temperatures that are at or beyond their upper optimum germination temperatures, so they should be vulnerable to future increases in temperature (Sentinella *et al.* 2020). Given adaptive differentiation in TPC parameters across species ranges (Fig. 1B), populations may vary in their vulnerabilities to changing climate, and TPCs provide information on tipping points beyond which population declines are likely (Angert *et al.* 2011; DeMarche *et al.* 2018). One research gap is to understand the roles of rapid evolution and acclimation of TPCs in buffering plant vulnerabilities to climate change. Second, there should be specialist–generalist trade-offs, such that a broad TPC comes at the cost of low maximum performance (Fig. 1C; Angilletta *et al.* 2003). There was a negative relationship between thermal performance breadth and maximum performance across genotypes of the annual plant *Mollugo verticillata* (Hereford 2017), suggesting that a ‘jack-of-all-temperatures is a master of none’ (Huey and Hertz 1984). Third, there should be associations between climate variability and niche breadth (climate variability hypothesis; Fig. 1D; Janzen 1967). In western North American monkeyflowers (*Mimulus*), species with broader TPCs experienced greater temperature variation across their ranges relative to species with narrower TPCs (Sheth and Angert 2014). Further, in response to 7 years of lower temperature seasonality compared to the historical mean, a population of scarlet monkeyflower (*Mimulus cardinalis*, a short-lived herbaceous perennial) evolved narrower thermal performance breadth (Wooliver *et al.* 2020). These two studies demonstrate that the climate variability hypothesis holds for changes in climate across both space and time, though support for this hypothesis based on plant TPCs is far from universal (Querns *et al.* 2022). Altogether, the hot–cold trade-off, the specialist–generalist trade-off and associations between climate variability and niche breadth lead to evolutionary divergence at multiple levels of biological hierarchy including genotypes within populations, populations within species and species within clades (Angert *et al.* 2011; Sheth and Angert 2014; Müller *et al.* 2017).

### Hypotheses on plasticity of TPCs

Thermal performance curves also shed light on two forms of plasticity. First, organisms may maintain high performance across a broad range of temperatures, indicated by a wide TPC (Reusch 2014). For example, many bromeliad species thermal performance breadths for germination are wide enough to encompass end-of-the-century temperature ranges (Müller *et al.* 2017), suggesting that they are an exception to the global trend of increasing vulnerability to climate change towards the tropics (Sentinella *et al.* 2020). Because TPCs were constructed from multiple seeds of only one to three individuals of each species, these thermal performance breadths represent plasticity. Such variation in TPC plasticity coincides with conclusions from animal literature that plasticity could rescue some, but not all species under climate change (Deutsch *et al.* 2008). Second, an individual’s critical thermal limits or thermal optimum may shift in response to prior thermal conditions (Fig. 1E), a process called ‘acclimation’ when involving long-term (days to months) exposure to warmer- or cooler-than-average temperatures (Gunderson *et al.* 2010; MacLean *et al.* 2019), and ‘hardening’ when involving short-term (seconds to minutes) exposure to more extreme temperatures (Neilson *et al.* 1972; Sinclair and Roberts 2005). Though studies on acclimation of fitness-based TPCs are limited, there is evidence that cold stratification of seeds can alter the shape of germination-based TPCs of the annual wildflower *Streptanthus tortuosus*, and this effect varies across populations (Gremer *et al.* 2020). Ultimately, variation in TPCs will be driven by a combination of genetic variation, trade-offs and plasticity (Sinclair *et al.* 2016), yet fitness-based plant studies are only beginning to scratch the surface of topics that require TPCs.

### Performance traits and timescales

Plant studies relying on TPCs to address key ecological and evolutionary hypotheses have used several proxies for individual or population mean fitness. While physiology-based TPCs that quantify performance at the subindividual level (e.g. leaf photosynthetic rates) rely on measurements of the same individuals across multiple temperatures and can take only a few minutes to quantify (e.g. Cunningham and Read 2003b), fitness-based TPCs require data collection on different individuals (often full siblings or genotypic clones) across multiple temperatures and require at least a few days or weeks to quantify. Though fitness-based TPCs require more time and effort, they offer a closer approximation of how plant populations would respond to climate change, especially since subindividual level traits can vary within an individual, at different times of day, etc. (Bassow and Bazzaz 1998). The most common trait in fitness-based plant TPCs is germination success, measured as percentage of seeds sown that germinate at a given temperature (e.g. Sentinella *et al.* 2020), followed by relative growth rate (e.g. Zhou *et al.* 2016), which has been measured over a range of timescales and life stages. For instance, in *M. cardinalis*, Wooliver *et al.* (2020) measured relative growth rates of ~3-week-old seedlings over a 1-week period, whereas Cunningham and Read (2003a) measured growth of year-old tropical tree seedlings across 16 weeks. Neither study assessed how well growth measured at these respective timescales and life stages represents growth at longer timescales or later life stages. Another timescale that could be important for plant TPCs is that of acclimation. For example, the cold stratification study of Gremer *et al.* (2020) quantified TPCs based on seeds that were either cold-stratified for 7 weeks or not cold-stratified. However, the literature lacks studies of acclimation effects on other fitness-based traits. Occasionally, studies have measured biomass (e.g. Hereford 2017) or survival (e.g. Chambers and Emery 2018), but rarely individual- or population-level reproductive traits such as flower, fruit or seed counts. One exception is Walters and Currey (2019), who measured the percentage of reproductive individuals at each temperature for different basil cultivars.

Given that these fitness proxies typically do not encompass the full life cycle, a key knowledge gap is whether fitness-based TPCs capture the relationship between temperature and the long-term growth rates of natural populations (Louthan *et al.* 2021; ‘Do fitness-based TPCs scale up to population-level thermal niches?’). A valuable starting point would be to evaluate the relationship between fitness proxies used in TPCs and measures of total individual fitness (e.g. fruit number or seed counts, if not lifetime fitness). For example, if a separate study of the same or related species shows that relative growth rate over a 7-day period is positively and strongly correlated with flower or fruit number, then we might have more confidence in this fitness proxy in a TPC study than in the absence of such information. Since different fitness proxies and life stages can yield different TPC shapes and parameters (e.g. Müller *et al.* 2018; Zotz *et al.* 2020), determining how sensitive TPCs are to choice of performance traits and life stages is ripe for study.

## Future Avenues for TPCs in Plant Ecology and Evolution

### Do populations and species harbour genetic variation for TPCs?

A fundamental question in evolutionary ecology is whether species accrue niche breadth via general-purpose genotypes with wide environmental tolerances and similar environmental optima, specialist genotypes with divergent environmental optima and narrow environmental tolerances or an intermediate combination of breadths and optima (Carscadden *et al.* 2020; Sheth *et al.* 2020). Thermal performance curves permit the examination of how niche breadth is partitioned among species in a clade, populations across species ranges and genotypes within populations (Roughgarden 1972; Carscadden *et al.* 2020; Fig. 1 in Sexton *et al.* 2017). This question has important implications for predicting how populations and species will respond to climate change. If species achieve broad TPCs via generalist populations, then any given population’s niche breadth approximates the species-level niche breadth, and incorporating population-level variation in TPCs may not yield further insights into range shift forecasts (Angert *et al.* 2011; Carscadden *et al.* 2020). In contrast, if species niches represent a collection of locally adapted populations, then range shift forecasts ignoring population-level variation in TPCs may underestimate any given population’s vulnerability to climate change (Kelly *et al.* 2012). Moreover, few studies have quantified genetic variation for TPCs, the raw material needed for adaptive evolution in response to changing temperatures, within populations (Huey *et al.* 2012).

Two studies have combined species distribution models (SDMs) with TPCs to investigate the hierarchical nature of niche breadth and predict how plant populations and species will respond to climate change. First, Hereford (2017) quantified TPCs for genotypes of *M. verticillata* in California (USA). Hereford (2017) compared a species-level SDM built from occurrence data to mechanistic SDMs built from genotype-level TPCs, and found that although genotypes varied in their degree of specialization (i.e. thermal performance breadth), there were positive correlations between suitability from the species-level SDM and mechanistic SDM predictions of biomass for each genotype across randomly drawn locations in California. Since these positive correlations were especially prevalent in genotypes with broad TPCs, Hereford (2017) concluded that many genotypes are generalists whose TPCs are likely sufficient for predicting the current and future distribution of *M. verticillata*. Second, using populations spanning the northern half of the geographic range of *M. cardinalis*, Angert *et al.* (2011) tested the hypothesis that a mechanistic SDM based on a species-level TPC would overestimate the amount of habitat projected to be climatically suitable in the future relative to mechanistic SDMs that account for intraspecific variation in TPCs. They built two types of species-level models using TPCs constructed from relative growth rates: a ‘species envelope’ model that used minimum and maximum thermal limits from the most thermally extreme population-level TPCs, and a ‘species average’ model derived from a species-level TPC fitting performance data from all populations. While the amount of projected suitable habitat was similar for the species envelope model and the population-level models, the species average model actually predicted less future suitable habitat than layered population-level models (Angert *et al.* 2011). This suggests that species-level TPCs can yield misleading inferences about vulnerability to climate change. Together, these studies support the notion that population-level, growth-based TPCs capture species-level niche breadth relatively well, but future work is needed to further examine how niche breadth is partitioned among species, populations and genotypes.

In addition to among-population variation in TPCs influencing vulnerability to climate change, genetic variation for TPC parameters within a population will ultimately determine a population’s ability to respond to temperature-mediated selection imposed by climate change. Though there are examples of animal populations that harbour significant additive genetic variance for TPC parameters (e.g. Latimer *et al.* 2011), we are unaware of any study that has quantified within-population genetic variation for TPC parameters in plants. Sheth and Angert (2014) examined whether *Mimulus* species with greater genetic variation for thermal tolerance have evolved broader TPCs. They were unable to fit TPCs to each genotype (represented by full-sibling seed families) to evaluate whether species with greater genetic variation for TPC parameters have evolved broader TPCs, because hierarchical TPC models that could simultaneously estimate multiple genotype-level TPCs from a population-level average TPC were not yet available. Rather, they focused on genetic variation for thermal reaction norms at the two lowest and the two highest experimental temperatures. They found that species with broader TPCs had greater among-genotype variation in slopes of reaction norms at both the cold (15–20 °C) and hot (40–45 °C) ends of the temperature gradient. Future work is needed to evaluate whether plant populations have sufficient genetic variation for TPC parameters to respond to climate change, and to further develop modelling approaches that can account for variation at multiple biological levels (e.g. Tittes *et al.* 2019).

### Do plant TPCs exhibit plastic responses to abiotic and biotic factors?

Plants experience a myriad of abiotic and biotic interactions, but how such interactions alter plant TPCs is a key knowledge gap that should be addressed in future work. Recent studies have shown that germination-based TPCs are shaped by abiotic factors including water availability (Fletcher *et al.* 2020), cold stratification (Gremer *et al.* 2020) and parental thermal environment (Singh *et al.* 2019). For example, drought can reduce the thermal optimum for germination in a population of the forest tree species *Eucalyptus tereticornis* (Kumarathunge *et al.* 2020). These findings emphasize the need to consider interactions between responses to multiple abiotic factors when forecasting plant responses to changing temperatures. How biotic interactions alter plant TPCs remains completely unexplored. For example, although virtually all plant species associate with endophytic microbes (Rodriguez *et al.* 2009; Smith and Read 2010) that mediate plant thermal limits for growth, survival and seed production (Redman *et al.* 2002; Hubbard *et al.* 2014; Acuña-Rodríguez *et al.* 2020) and geographic distributions (Afkhami *et al.* 2014; Van Der Heijden *et al.* 2018), nothing is known about their influences on plant TPCs.

**Figure 2.**
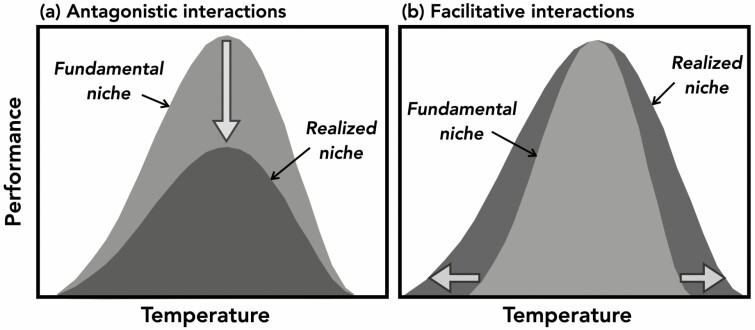
Prediction of how plant TPCs should be mediated according to the stress-gradient hypothesis. If (A) antagonistic interactions that reduce plant performance dominate at benign temperatures and (B) facilitative interactions that enhance plant performance dominate at extreme temperatures, realized TPCs should respectively be shorter and wider than fundamental TPCs.

Thermal performance curves are a powerful tool for evaluating how fundamental thermal niches differ from realized thermal niches. If, according to the stress-gradient hypothesis, antagonistic interactions dominate at benign temperatures and facilitative interactions dominate at extreme temperatures, realized TPCs should be shorter (Fig. 2A) and wider (Fig. 2B) than fundamental TPCs. Even though antagonists (pathogens and competitors) can reduce the thermal performance breadths of animals in the absence of facilitative interactions (Tsai *et al.* 2020; Hector *et al.* 2021), the stress-gradient hypothesis suggests that facilitative interactions would overpower these negative effects of antagonists at thermal extremes. Further, because warming often increases drought intensity, facilitative microbes that increase drought tolerance (Kivlin *et al.* 2013) may be more beneficial to plants under future climate change, potentially elevating performance under increasing temperatures relative to a no-microbe control. In this case, we may underestimate plant thermal performance breadth if we do not consider plant–microbe interactions. Still, plant responses to facilitative interactions in stressful environments depend on developmental stage (David *et al.* 2020), so future studies of biotic effects on plant TPCs should consider estimating TPCs at more than one life stage. Further, there may be geographic variation in effects of facilitative interactions on plant TPCs. For example, we would expect extension of lower thermal limits in high-latitude populations, and upper thermal limits in low-latitude populations, due to co-adaptation to cooler vs. warmer temperatures; or greater microbial-induced plasticity in equatorial populations due to the greater importance of biotic interactions at lower latitudes (Darwin 1859; Paquette and Hargreaves 2021). If the stress-gradient hypothesis holds for plant TPCs, accounting for facilitative and antagonistic interactions will be critical for predicting responses to climate change.

### Do fitness-based TPCs scale up to population-level thermal niches?

**Figure 3.**
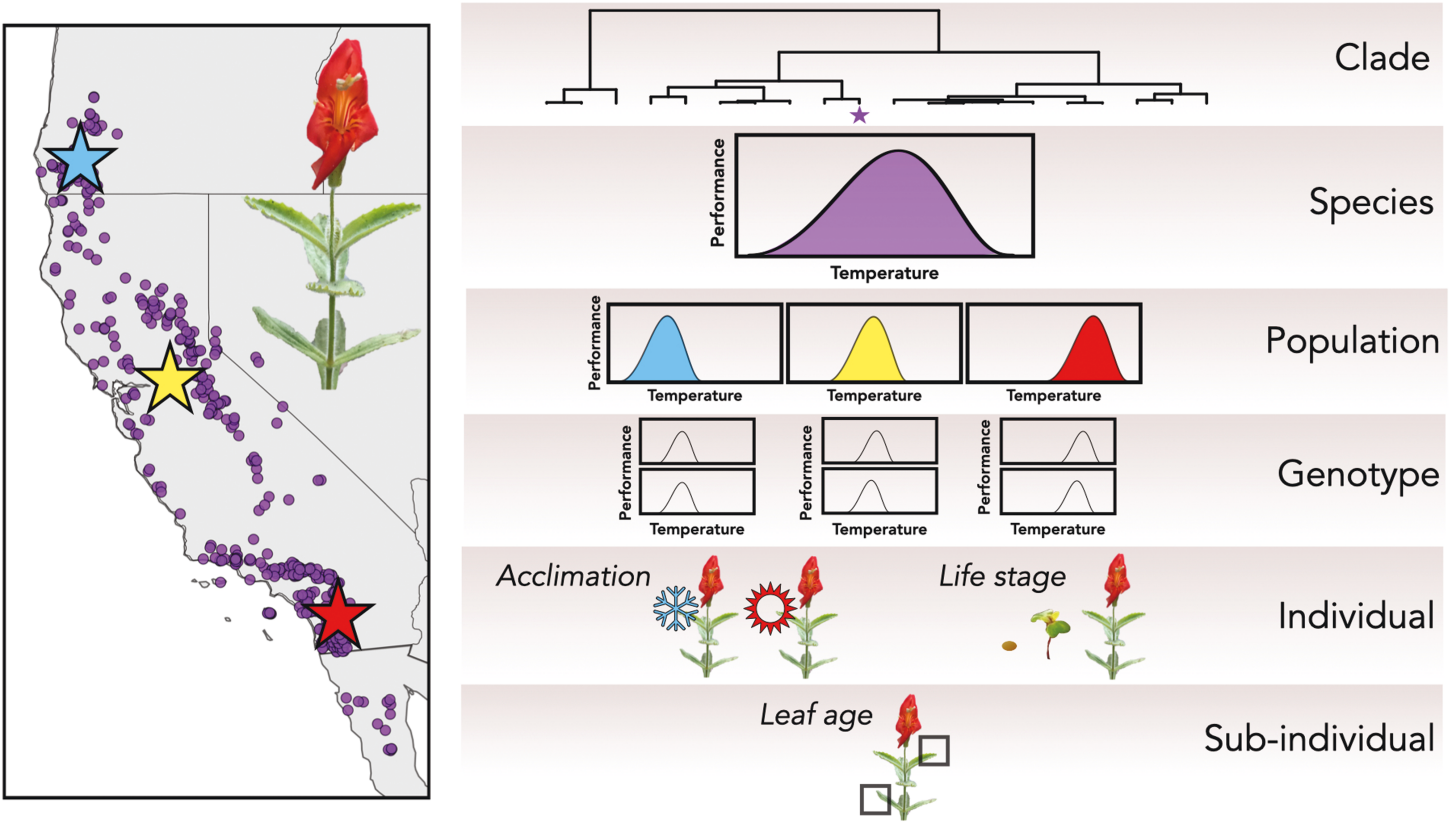
Levels of biological hierarchy relevant for plant TPC studies. Consider a plant species, for example *Mimulus cardinalis*, whose range in western North America is shown based on herbarium specimens (Angert *et al.* 2018). Populations across the geographic range may contribute to a broad species-level TPC, with evolutionary divergence in TPCs across populations consistent with local adaptation. Genotypes (represented by genetic clones or seed families) within each population contribute to the population-level TPCs. However, TPCs of individual plants within each genotype may vary through factors such as space and time via thermal acclimation, leaf age and life stage. These levels of hierarchy can be nested into deeper evolutionary clades. Though we do not address them in this viewpoint, TPCs can be built from repeated measurements of photosynthesis from the same leaf or branch across a temperature gradient. Rather, we address TPCs at the genotype level and above, which are constructed from independent measurements of growth or fitness data from different individuals exposed to different temperatures.

In the strictest sense, the thermal niche represents the set of temperatures across which population growth rate (*λ*) is ≥1, indicating demographic stability or growth, whereas a TPC describes a performance metric for a biological entity (a subindividual, individual, genotype, population or species; Fig. 3) across a range of temperatures (Hutchinson 1957; Huey and Stevenson 1979). Because *λ* is equivalent to the mean fitness of a population, the thermal niche represents the temperatures across which individuals in a population can survive, grow and reproduce. Consequently, if the goal of a TPC study is to quantify the thermal niche, the gold standard for measuring performance is a composite measure of total fitness, rather than metrics related to growth or single fitness components. Recent studies have sought to quantify the thermal niches of populations or species through TPCs with the goals of predicting sensitivity to future climate change (Müller *et al.* 2017) and forecasting range shifts (Angert *et al.* 2011). Ideally, the critical thermal maximum in such studies would represent a temperature that, if reached in a future climate change scenario, would cause population declines (*λ* < 1). While extreme temperature events may drive short-term acclimation responses at the subindividual level (Geange *et al.* 2021), mean temperature experienced over a generation should drive evolution of fitness-based TPCs across generations.

Previous work has estimated TPCs using population growth rate as the response variable in short-lived organisms including *Daphnia* (Bruijning *et al.* 2018) and numerous insect species (Deutsch *et al.* 2008; Rezende and Bozinovic 2019). Such studies do not exist for plants due to the logistical challenges associated with quantifying vital rates such as survival probability, growth, flowering probability and fruit number, some of which cannot be accurately measured in a lab setting, particularly for long-lived organisms and outcrossers that rely on pollinators. However, the closer performance metrics are to approximating fitness, the more the TPC will approach the true thermal niche. Recent studies have quantified fitness curves for annual plant systems across soil moisture gradients (Tittes *et al.* 2019; Pearse *et al.* 2020), bolstering their promise for approximating the thermal niche. For systems where it is not practical to estimate fitness components, much less lifetime fitness, the best approximation of the thermal niche is through TPCs for multiple life stages (Müller *et al.* 2018).

## Conclusions

Thermal performance curves are an ideal tool for fundamental and applied research in plant ecology and evolution. Here, we have described plant TPC studies that have tested hypotheses about plant adaptation and plasticity, with important implications for predicting responses to climate change. Several hypotheses, however, remain untested, including those about the roles of genetic variation in the evolution of niche space at the population level and beyond, controls of biotic interactions on realized niche space, and whether fitness-based TPCs determined in experimental settings can be scaled up to represent population-level niche space *in situ*. Ultimately, we hope that this viewpoint paves the way for including plants in future syntheses of TPCs and deepening our understanding of the ecological and evolutionary determinants of plant responses to climate change.

## Data Availability

There are no data associated with this manuscript.

## Notes

**Populations & Communities. Chief Editor**: Jean Burns
